# Endocrine responses to low‐load blood flow restricted and high‐load resistance exercise in well‐trained males

**DOI:** 10.14814/phy2.70455

**Published:** 2025-07-09

**Authors:** Drake A. Eserhaut, Joseph M. DeLeo, Jessica A. Provost, Andrew C. Fry

**Affiliations:** ^1^ Jayhawk Athletic Performance Laboratory—Wu Tsai Human Performance Alliance University of Kansas Lawrence Kansas USA; ^2^ Female Athlete Program—Wu Tsai Human Performance Alliance Boston Children's Hospital Boston Massachusetts USA

**Keywords:** catecholamines, cortisol, growth hormone, testosterone, vascular occlusion

## Abstract

The present study compared acute testosterone (T), cortisol (C), epinephrine (EPI), norepinephrine (NE), and 22 kDa growth hormone (GH‐22 kDa) responses following low‐load resistance exercise with blood flow restriction (LL‐BFR) and traditional high‐load resistance exercise (HL‐RE). Twelve resistance‐trained men performed bouts of LL‐BFR (30%1RM) and HL‐RE (70%1RM), each consisting of four sets of bilateral seated leg extensions taken to momentary task failure with 60 s rest periods. A randomized crossover design was used with time of day matched within‐subjects. Upon arrival between 1200 and 1800, 24 h dietary recalls were performed with post‐exercise blood samples obtained within 60 s (IP) and 5 min post‐exercise (+5 min) via intravenous cannulation. Greater total repetitions (*d* = 2.37, *p* < 0.001) and less volume‐load (*d* = 2.86, *p* < 0.001) were performed during LL‐BFR. No Condition × Time interaction effects were found for any hormonal analyte measured (*p* > 0.05). Both LL‐BFR and HL‐RE elevate the potent β_2_ adrenergic receptor (β_2_AR) agonist EPI (IP: 1.29 ± 0.44 and 1.35 ± 0.60 nmol·L^−1^, respectively), and the androgenic steroid T (+5 min: 27.4 ± 12.9 and 29.0 ± 14.3 nmol·L^−1^, respectively). Thus, acute skeletal muscle β_2_AR phosphorylation may be comparable between conditions. When lower resistance exercise intensities (e.g., 30% 1RM) are desired, athletes may perform LL‐BFR in place of HL‐RE and experience no statistical difference in acute endocrine responses.

## 1 INTRODUCTION

The addition of mechanically induced vascular occlusion, also known as blood flow restriction (BFR), during low‐load resistance exercise (e.g., 30% one‐repetition maximum [1RM]) has been utilized as an alternative training method for garnering skeletal muscle hypertrophic and maximal strength adaptations comparable to those experienced following high‐load resistance exercise (e.g., 70% 1RM) (Grønfeldt et al., 2020; Luebbers et al., 2014; Reece et al., 2023). Such protocols may permit athletes to train using lower absolute loads and experience comparable, or slightly greater, muscle hypertrophy and maximal strength adaptations. Such outcomes may be advantageous during phases of training where in‐season competition demands are high and practitioners seek to limit noncompetition‐related training demands such as during periods of de‐loading or tapering (Davids et al., 2023; Lorenz et al., 2021). When BFR cuffs are worn continuously during resistance exercise, the rate at which the microcapillaries within exercising skeletal muscle tissue resaturate with oxygen‐bound hemoglobin (SmO_2_resat) during interset rest periods appears to be significantly blunted, which may be one effect responsible for the application of BFR expediting the onset of local muscle fatigue during exercise and permitting the use of lower absolute loads (Eserhaut et al., 2024). Further, should LL‐BFR be capable of inducing significant amplifications in numerous hormonal concentrations during acute training bouts, when applied regularly throughout structured training regimens, slightly more favorable hypertrophic adaptations may be garnered via repeated exposure to acute elevations in endogenous hormonal concentrations.

The physiological roles of classically anabolic hormones such as testosterone (T) and growth hormone in skeletal muscle hypertrophy have been debated (West & Phillips, 2012); however, their involvement in vital skeletal muscle metabolic and anti‐proteolytic processes is well founded (Hooper et al., 2017; Hymer et al., 2020). Randomized placebo‐controlled trial data show the suppression of endogenous T via administering the gonadotropin‐releasing hormone (GnRH) analog goserelin, which impedes luteinizing hormone production and thus testicular T production, significantly blunted increases in lean mass, markedly reduceed gains in maximal strength, and increased body fat percentage following 8 weeks of resistance training (Kvorning et al., 2006). Further, the analyte growth hormone exists as a superfamily of more than 100 isoforms, each with varying molecular masses, and thus differing physiological and immunological functions (Baumann, 1991; Pierce et al., 2020). The 191 amino acid isoform of growth hormone possessing a molecular mass of 22 kilodaltons (GH‐22 kDa) is by far the most abundant growth hormone isoform and is detected in the majority of commercially available growth hormone immunoassays (Wood, 2001). GH‐22 kDa has been shown to increase significantly following lower body LL‐BFR in recreationally trained males (Madarame et al., 2010), with the growth hormone family collectively contributing to numerous physiological processes, such as substrate mobilization, protein synthesis, and anabolism (Nindl et al., 2003). Thus, resistance training protocols capable of inducing acute amplifications in endogenous T and GH‐22 kDa concentrations may permit subtly greater adaptations if employed routinely throughout moderate‐to‐long term training regimens.

In highly‐trained athletic populations, low‐load resistance exercise performed with BFR has been shown to significantly elevate concentrations of free‐testosterone in saliva in a large cohort of Division II American football athletes (Luebbers et al., 2025). Further, relative to a volume‐matched bout of low‐load bilateral seated leg extensions performed without BFR, the addition of BFR cuffs with pressure applied to both proximal thighs continuously across all sets significantly elevated acute post‐exercise salivary cortisol responses, and the enzymatic proxy for sympathetic nervous system activity alpha‐Amylase in well‐resistance trained men, indicative of the addition of BFR during low‐load resistance exercise yielding greater hypothalamic–pituitary adrenal (HPA) axis activity than performing the same volumes of resistance exercise without the cuffs (Eserhaut et al., 2024). Given BFR's ability to augment acute salivary hormone responses to resistance exercise, paired with reports of LL‐BFR inducing significant elevations in blood‐derived concentrations of cortisol (C), norepinephrine (NE), and GH‐22 kDa in untrained and recreationally active individuals (Iida et al., 2007; Madarame et al., 2010; Sharifi et al., 2020), further endocrine‐focused investigations in individuals of a higher resistance training status are of particular importance should LL‐BFR be sought for use with healthy athletic populations.

Specific interest lies in the potent fight‐or‐flight hormone epinephrine (EPI), as little data exist on this analyte within the context of BFR training. Given the pronounced involvement of EPI in numerous metabolic and anti‐proteolytic functions within skeletal muscle (Graça et al., 2013; Long et al., 2004; Nicoll et al., 2019), data on LL‐BFR's impact on this sympathetic neurohormone would aid in the collective understanding of BFR's physiological effects during resistance exercise. Additionally, the role of EPI's target skeletal muscle receptor, the β_2_ adrenergic receptor (β_2_AR), has received attention in the current body of resistance training‐related skeletal muscle physiology literature as one of many potential hypertrophic signaling pathways (Roberts et al., 2023). However, most studies reporting on the catecholamine responses to various exercise and passive BFR bouts report on the catecholamine norepinephrine (NE) (Loenneke et al., 2012). Importantly, this adrenergic receptor‐mediated signaling is dependent on the beta‐adrenoreceptor subtype. NE preferentially binds to β_1_ adrenergic receptors (β_1_AR), which are primarily located within cardiac muscle tissue (Frielle et al., 1988). Skeletal muscle, however, contains predominantly the β_2_ subtype of adrenergic receptors, with only about 7%–10% being of the β_1_ subtype (Williams et al., 1984). Therefore, with respect to the hormonally mediated signaling of hypertrophic and metabolic pathways within skeletal muscle, EPI is the primary catecholamine of interest due to its far greater binding affinity to the β_2_ARs on skeletal muscle cells (Graça et al., 2013; Nicoll et al., 2023; Sterczala et al., 2017).

Thus, the purpose of this study was to investigate numerous acute hormonal responses to acute bouts of LL‐BFR and HL‐RE in well‐resistance trained men. Further, we aimed to provide data on the metabolic analyte blood lactate (BLa) and local skeletal muscle oxygen resaturation kinetics across protocols. We hypothesized that LL‐BFR would induce similar or slightly greater responses in all hormonal analytes in comparison to the bout of HL‐RE, with subjects experiencing marked reductions in interset SmO_2_resat during LL‐BFR.

## 2 MATERIALS AND METHODS

### 2.1 Experimental approach to the problem

A within‐subjects randomized crossover design with repeated measures was used, with 1‐week of recovery between each acute resistance exercise bout. The resistance exercise testing sessions (LL‐BFR and HL‐RE) were randomized between subjects and counter‐balanced such that six out of the twelve subjects performed LL‐BFR in the first testing session, and six out of twelve performed HL‐RE in the first testing session. On the day of the first testing session, subjects arrived at the laboratory between the hours of 1200 and 1800. During this time of passive rest, 24 h dietary recalls were performed by a registered dietitian. After which, reports of subjects' 24 h dietary information were sent to them with instructions to mimic their macronutrient consumption and fluid intakes prior to arrival for the final resistance exercise testing session to the best of their ability. The time of day for testing was matched within‐subjects, such that were a given individual was scheduled for the first resistance exercise testing session at 1400, they also completed the second and final resistance exercise testing session at 1400.

### 2.2 Subjects

Twelve (*n* = 12) well resistance trained male subjects were recruited for this study and refrained from strenuous physical activity at minimum of 48 h prior to the baseline strength assessments and both acute resistance exercise bouts. They also withdrew from alcohol, tobacco, and any form of caffeine intake 24 h prior to testing. Subjects were required to have a barbell back squat one‐repetition maximum (1RM) equal to or greater than 1.5× their bodyweight and self‐reported engagement in a minimum of 2 years or more of routine lower body resistance exercise with consistent performance of lower body resistance exercise in the 6 months preceding study participation. Subjects were also required to be free from any musculoskeletal injuries and neurological, cardiovascular, or metabolic disorders. All subjects were thoroughly briefed on study procedures prior to signing an informed consent statement as approved by the University of Kansas' institutional review board (STUDY#00151133) in accordance with the 1964 Helsinki Declaration.

### 2.3 Procedures

#### 2.3.1 Baseline assessments

Subjects first reported for an initial pre‐study assessment of baseline physical, health, and performance characteristics. After sitting passively for informed consenting procedures, resting upper‐arm blood pressure was measured using an automated device (Omron BP7450, Omron Healthcare, Kyoto Japan). Height was then measured using a wall‐mounted stadiometer. Body mass was assessed, as well as estimates of body composition via bioelectrical impedance analyses (BIA) (SECA mBCA 515, Hamburg, Germany). Subjects then sat on a plinth table with their left leg set in a state of passive full extension, after which 1/3 of the distance between the anterior superior iliac spine and the proximal, lateralmost tip of the patella was measured via Gulick tape measure for the assessment of skinfold thickness via calibrated calipers (Harpenden, Nutriactiva, England, United Kingdom) at the site of the near‐infrared spectroscopy (NIRS) sensor. Proximal thigh circumferences were also assessed immediately inferior to the gluteal fold. While in a seated position, subjects were equipped with 10 cm wide contoured, single‐chambered, pneumatic blood flow restriction cuffs (Smart Cuffs v4.0, Smart Tools Plus, Ohio, United States) for the assessment of arterial occlusion pressure (AOP) for each leg. AOP was assessed via doppler ultrasound by monitoring the pulse of the posterior tibial artery during slow graded increases in pressure until the audible sounds of the arterial pulse ceased. Following pulse cessation, BFR cuff pressure was released slowly until the pulse returned, with the pressure at which return occurred recorded as the AOP for that leg (de Queiros et al., 2024). For 2 subjects, the BFR cuffs used were unable to be inflated to a pressure that achieved full AOP (system max = 250 mmHg), and thus, 300 mmHg was used for their AOPs. Subjects then transitioned to a self‐selected dynamic warm‐up, after which maximal barbell back squat 1RM was determined using prior methods (Kraemer et al., 2006) as an additional determinant of resistance training status. A minimum of 10 min passive rest was then allotted, followed by bilateral seated leg extension 1RM testing (Eserhaut et al., 2024). All 1RM testing was administered by a National Strength and Conditioning Association (NSCA) certified strength and conditioning specialist. Additionally, data from our lab on a separate cohort of resistance trained men (*n* = 16) yielded high test–retest reliability for 1RM testing evidenced by excellent coefficients of variation (CV) and two‐way mixed‐effects model intra‐class correlation coefficients (ICC_2,1_) for the back squat (CV = 1.5%; ICC_2,1_ = 0.97) and bilateral seated leg extension (CV = 2.7%; ICC_2,1_ = 0.97). Relative maximal back squat strength was calculated as the ratio between the raw 1RM number in kilograms divided by each subject's body mass in kilograms. All aforementioned data is provided in Table 1.

**TABLE 1:** Participant anthropometric, health, and performance characteristics.

	Mean ± SD	Range
No. of participants	12	
Age, yr	24.1 ± 3.6	19.0–31.0
Height, cm	180.4 ± 4.1	174.0–186.7
Body mass, kg	88.6 ± 13.8	75.6–120.7
Fat‐free mass, kg	71.4 ± 4.9	65.6–81.8
Body fat percentage, %	17.5 ± 7.0	9.6–32.2
SBP, mmHg	128.4 ± 11.5	105–152
DBP, mmHg	71.2 ± 9.1	57.7–90.5
Proximal thigh circumference, cm	63.0 ± 4.5	56.5–71.1
AOP, mmHg	251.1 ± 26.4	200.0–300.0
80% AOP, mmHg (for LL‐BFR condition)	200.9 ± 21.1	160.0–240.0
SQ 1‐RM, kg	162.5 ± 40.6	120.5–263.6
Relative 1‐RM SQ strength, kg•kg^−1^bodyweight	1.8 ± 0.3	1.5–2.5
Seated leg extension 1‐RM, kg	55.4 ± 5.1	42.0–63.6

*Note*: Data presented as x̄±SD. Relative strength calculated as barbell back squat 1‐RM (kg) divided by bodyweight (kg).

Abbreviations: 1‐RM, one‐repetition maximum; AOP, arterial occlusion pressure; DBP, diastolic blood pressure; kg, kilograms; LL‐BFR, low‐load blood flow restriction training; SBP, systolic blood pressure; SQ, barbell back squat; yr, years.

#### 2.3.2 Acute resistance exercise bouts

Subjects first completed a 5 min warm‐up on a stationary cycle ergometer (Monark Ergomedic 817E, Monark Exercise AB, Vansbro Sweden) at a self‐selected low intensity prior to transitioning to the acute bout of resistance exercise. The LL‐BFR protocol consisted of subjects standing and having the BFR cuffs affixed bilaterally on both thighs immediately below the gluteal fold, secure enough to remain in place without falling while still permitting rotation around the thigh. Subjects then sat in the weight stack style seated leg extension machine (Universal Conditioning Equipment, Iowa, United States) and rested both legs in passive extension on a chair. The BFR cuffs were then inflated to 80% AOP one at a time, with the right cuff being inflated first, followed by the left cuff. This sequencing was done to avoid markedly altering pre‐exercise NIRS data from the left vastus lateralis. The prescribed BFR cuff pressure of 80% AOP was chosen as an aggressive, yet safe, upper limit for lower body LL‐BFR (de Queiros et al., 2024), with 80% AOP shown to markedly blunt interset SmO_2_resat in the vastus lateralis during continuous application (Eserhaut et al., 2024). Using 30% 1RM, subjects then performed bilateral seated leg extensions for four sets to momentary task failure at a cadence of 60 beats per minute, corresponding to a 1 s concentric and a 1 s eccentric phase, with sets terminated when either repetition cadence was broken or full knee extension was not achieved for two consecutive repetitions. Interset rest periods were 1 min in duration. The BFR cuffs were worn continuously throughout the entire protocol, with deflation and removal occurring immediately after achieving momentary task failure on set 4. For the HL‐RE protocol, bilateral seated leg extensions were also performed to momentary task failure using the same 1 min inter‐set rest periods, repetition cadence, and set termination criteria, but with 70% 1RM and no BFR cuffs.

#### 2.3.3 Near‐infrared spectroscopy data acquisition

A wearable NIRS device (MOXY Monitor; Fortiori Design LLC, Minnesota, United States) was affixed on the left vastus lateralis (VL) at 1/3 the distance between the anterior superior iliac crest and the proximal, lateral‐most patellar tip with CoverRoll stretch tape for all subjects (Feldmann et al., 2019). Care was taken to cleanse the skin with an alcohol wipe prior to attaching the NIRS device along with the provided light shield. The NIRS device was set to sample at a rate of 1 Hz, corresponding to the manufacturer's “medium” setting. All NIRS data was downloaded from the VO2 Master cloud software as Excel files prior to analysis. Data for SmO_2_ resaturation rates (SmO_2_resat) were calculated as the positive linear slope of the first 30 s of SmO_2_ data during each 1 min interset rest period (Eserhaut et al., 2024, 2025). SmO_2_resat data from sets 1, 2, and 3 were averaged for later between‐condition comparative analyses (mean SmO_2_resat). SmO_2_resat following the conclusion of set 4 was not included as BFR cuff removal occurred immediately following subjects achieving momentary task failure.

#### 2.3.4 Blood sampling and processing procedures

Upon arrival, one of two nationally certified phlebotomists inserted an intravenous cannula into an antecubital vein. The cannula was attached to a 0.9% saline drip with a 3‐way stopcock apparatus to maintain patency and permit rapid post‐exercise blood sampling. Subjects then sat passively for 30 min to return to a psychophysiological state of homeostasis, after which a resting blood sample was obtained (PRE). Immediately upon completion of each resistance exercise bout, a post‐exercise blood sample was collected within 60 s (IP), followed by the final sample at +5 min (+5 min). A 3 mL syringe was used to extract 1–2 mL of blood (with saline from the cannula) for discard prior to the acquisition of all blood samples. Approximately 6 mL of blood was collected into an untreated Vacutainer™ for serum and a 10 mL Vacutainer™ for plasma. Immediately following sample acquisition, untreated whole blood was analyzed for BLa concentrations using the Lactate Pro 2 handheld analyzer (Arkay, Kyoto, Japan), which possesses strong intra‐device reliability (Crotty et al., 2021). Serum was then analyzed for hemoglobin concentrations using microcuvettes and the HemoCue Hb 201+ analyzer (HemoCue, Ängelholm, Sweden) with hematocrit also determined via microcapillary tube centrifugation, permitting the calculation of plasma volume shifts during exercise (Dill & Costill, 1974). Serum was allowed to clot at room temperature for a minimum of 30 min, after which Vacutainer™ tubes were centrifuged for 15 min at 1500RPM. Serum and plasma were then aliquoted into Eppendorf tubes and stored at −80°C for later analysis.

#### 2.3.5 Enzyme‐linked immunosorbent assays

Serum concentrations of total T, C, and GH‐22 kDa were analyzed in duplicate using commercially available enzyme‐linked immunosorbent assay (ELISA) kits (Cat No.: TST31‐K01; COR31‐K01; HGH31‐K01; Eagle Biosciences, New Hampshire, United States), with absorbances read at a wavelength of 450 nM using a microtiter plate photometer (accuSkan FC, Fisher Scientific, Massachusetts United States). Plasma concentrations of the catecholamines EPI and NE were analyzed in duplicate using a commercially available ELISA kit (Cat No.: IB89549R; IBL‐America, Minnesota, United States). All analytes were assayed by a single laboratory technician, with all samples from each subject run within the same microtiter plate. Intra‐ and inter‐assay CVs were <5% and <6%, respectively, for all analytes. All samples were thawed only once before analysis. Hormonal values were not corrected for plasma volume shifts as we deemed it important to know the concentration of hormone that target tissues are exposed to acutely post‐exercise regardless of the means by which the change in concentration occurred (Hackney et al., 1995; Kraemer et al., 1990).

### 2.4 Statistical analyses

All data were first evaluated for normality using Q‐Q plots and the Shapiro–Wilk statistic. NE expressed moderate‐to‐strong positive skews in both conditions at all three sampling times (PRE, IP, +5 min) (Skewness: HL‐RE = 2.19, 2.07, 2.30; LL‐BFR = 0.93, 1.98, 1.98; Shapiro–Wilk: *p* < 0.05). Thus, a log10 transformation was applied to NE concentrations prior to analysis, yielding normal data distributions (Skewness: HL‐RE = 1.09, 1.02, 1.11, LL‐BFR = 0.54, 0.57, 0.54; Shapiro–Wilk: *p* > 0.05). GH‐22 kDa also expressed moderate‐to‐strong positive skews in both conditions at all three sampling times (PRE, IP, +5 min) (Skewness: HL‐RE = 2.70, 2.37, 2.08; LL‐BFR = 1.57, 1.81, 2.44; Shapiro–Wilk: *p* < 0.05). Thus, a log10 transformation was applied to GH‐22 kDa prior to analysis as well, yielding normal data distributions (Skewness: HL‐RE = −0.52, 0.67, 0.43, LL‐BFR = −0.36, 0.35, 0.11; Shapiro–Wilk: *p* > 0.05). All visualizations display untransformed data. Further, the T to C ratio (T/C) was curated by dividing serum T concentrations by the corresponding C concentrations.

Volume‐load was calculated as the mass in kilograms multiplied by the total number of repetitions completed. The rate of SmO_2_resat during each interset rest interval was averaged, yielding a mean SmO_2_resat metric representative of local muscle oxygen reperfusion rates across both protocols for each individual. Resistance exercise performance variables (total repetitions and volume‐load), as well as mean SmO_2_resat, were compared using paired samples *t*‐tests with Cohen's *d* effect sizes used to interpret the magnitude of between‐condition differences with the following thresholds: weak <0.2, weak‐to‐moderate 0.2–0.4, moderate = 0.4–0.65, moderate‐to‐strong = 0.65–0.7, and strong >0.8 (Rubin, 2012). Changes in whole blood descriptive variables (hematocrit and hemoglobin), along with metabolic (BLa) and endocrine (T, C, T/C, GH‐22 kDa, EPI, NE) analytes were assessed using 2 × 3 Condition [LL‐BFR, HL‐RE] × Time [PRE, IP, +5 min] repeated measures analyses of variance (RMANOVA's), with partial eta‐squared ($\eta_{p}^{2}$) used for effect sizes. Plasma volume changes were assessed via a 2 × 2 Condition [LL‐BFR, HL‐RE] × Time [IP, +5 min] RMANOVA. Based on suggestions from Richardson, 2011, $\eta_{p}^{2}$ = 0.01 was considered a small effect, $\eta_{p}^{2}$ = 0.06 a medium effect, and $\eta_{p}^{2}$ = 0.14 a large effect (Richardson, 2011). Statistically significant interaction effects were followed up with Bonferroni corrected post hoc tests. Statistical inferences for all analyses were made using an a priori alpha level of *p* < 0.05. All statistical analyses were performed in Jeffrey's Amazing Statistics Program (JASP: version 0.18.3), with data visualizations created in R Studio (version 1.4.1106).

## 3 RESULTS

### 3.1 Resistance exercise performance

**FIGURE 1:**
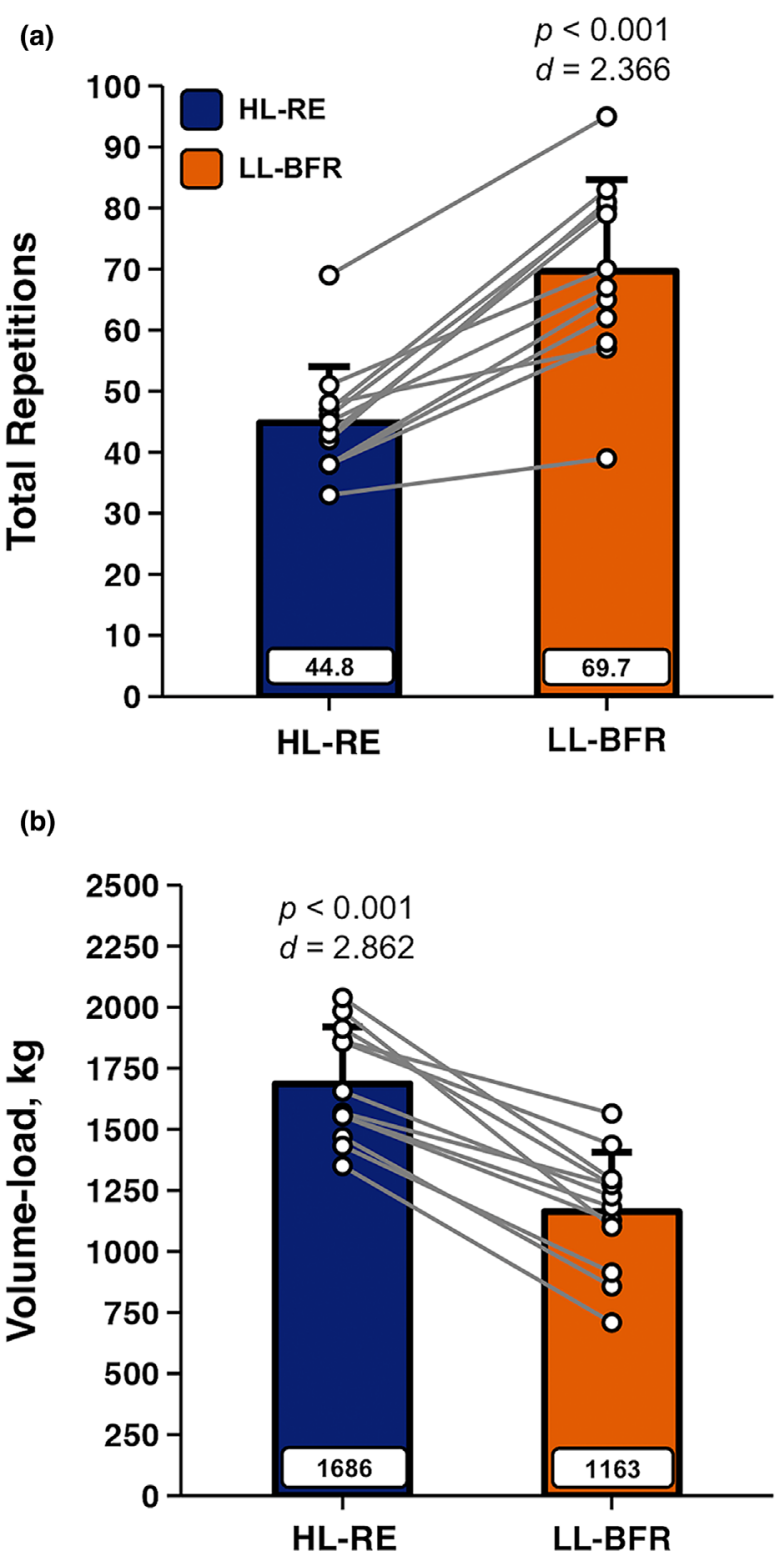
Data are presented as Mean ± SD. Comparisons of (a) total repetitions performed to momentary task failure across four sets, along with (b) volume‐load (total repetitions × load in kg) during high‐load (HL‐RE) and low‐load blood flow restricted (LL‐BFR) resistance exercise in well‐trained men (*n* = 12). Cohen's *d* effect sizes and *p* values from paired‐samples *t*‐tests are overlaid, with mean values displayed in rectangular data labels.

The mean ± SD (range) for the number of repetitions completed on sets 1, 2, 3, and 4 during HL‐RE were 21 ± 5 (12–28), 11 ± 3 (7–16), 8 ± 3 (5–15), and 7 ± 2 (5–12). The mean ± SD (range) for the numbers of repetitions completed on sets 1, 2, 3, and 4 during LL‐BFR were 39 ± 9 (25–52), 14 ± 3 (10–18), 9 ± 3 (3–13), and 8 ± 4 (1–15). Total repetitions completed were significantly greater during LL‐BFR as shown in Figure 1, panel a. Inversely, total volume‐load was significantly greater during HL‐RE, as shown in Figure 1, panel b.

### 3.2 Skeletal muscle oxygen resaturation kinetics

**FIGURE 2:**
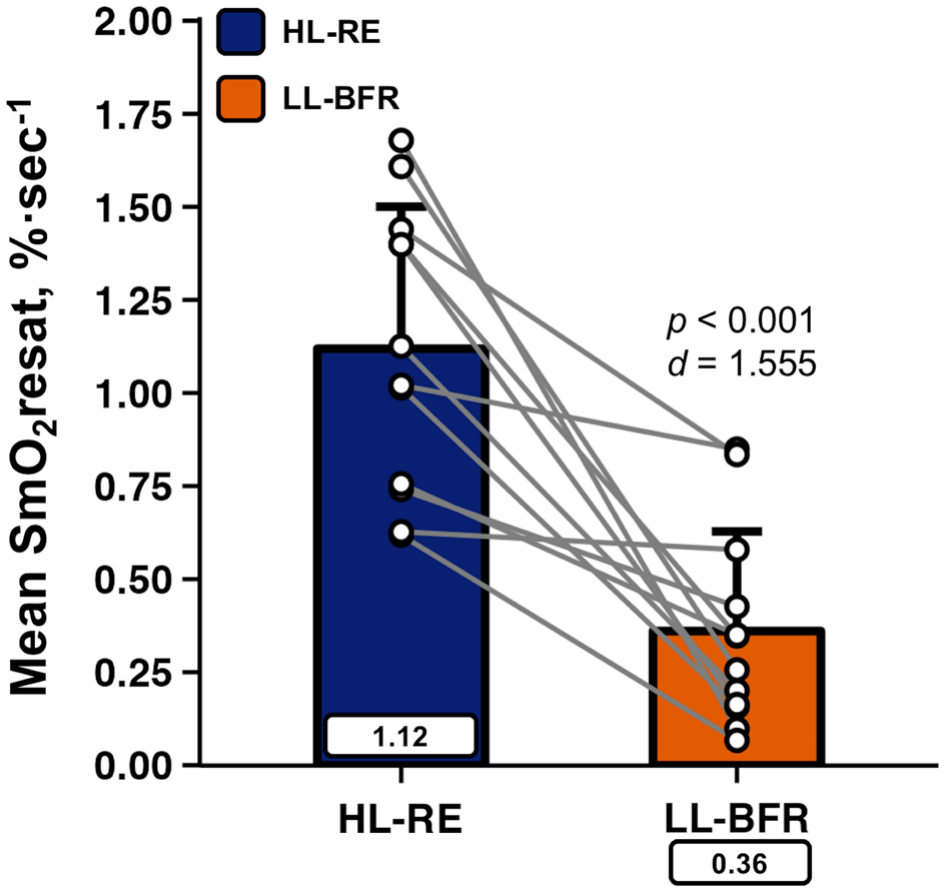
Data are presented as Mean ± SD. Comparisons of mean skeletal muscle oxygen resaturation rates (mean SmO_2_resat) across high‐load (HL‐RE) and low‐load blood flow restricted (LL‐BFR) resistance exercise protocols in well‐trained men (*n* = 12). Cohen's *d* effect size and resultant *p* value from the paired‐samples *t*‐test is overlayed, with mean values displayed in rectangular data labels.

The LL‐BFR protocol significantly blunted mean SmO_2_resat in comparison to the non‐BFR HL‐RE protocol, as shown in Figure 2.

### 3.3 Hemoglobin, hematocrit, plasma volume shifts, and blood lactate

**FIGURE 3:**
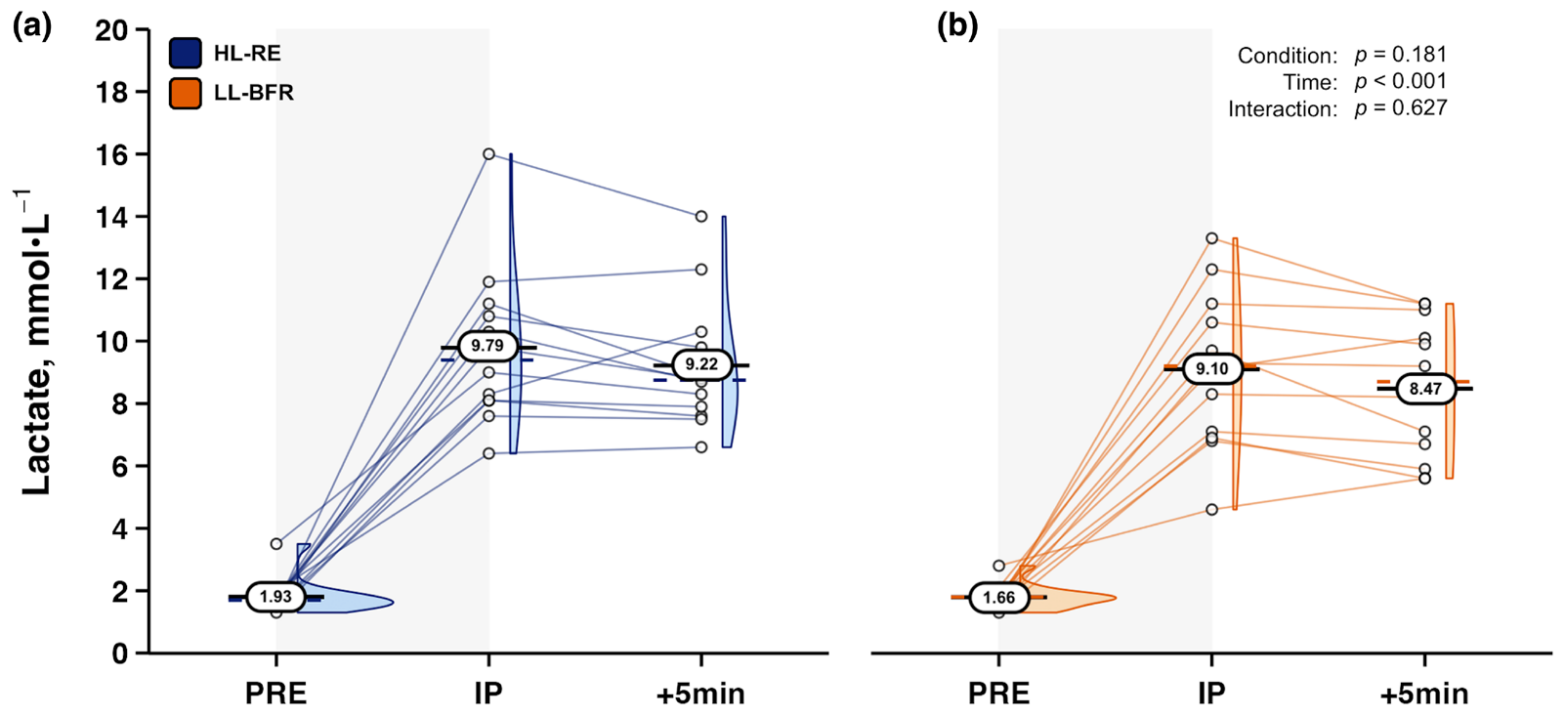
Serum blood lactate responses to (a) high‐load resistance exercise (HL‐RE) and (b) low‐load blood flow restricted resistance exercise (LL‐BFR) in well‐trained men (*n* = 12). Oval data labels with horizontal lines = means; Dashed colored lines = medians; Histograms at each time point are also provided. *p* values from 2 × 3 (Condition × Time) repeated‐measures ANOVA are overlayed for main effects and interaction effects. +5 min, five minutes post‐exercise; IP, immediately post‐exercise; PRE, pre‐exercise.

For the hematologic measure hemoglobin (Hb), there was no Condition × Time interaction (*p* = 0.736) or main effect for Condition (*p* = 0.562). There was, however, a main effect for Time (*F*_[2,22]_=76.504, *p* < 0.001, $\eta_{p}^{2}$ = 0.874). Hematocrit (Hct) also displayed no Condition × Time interaction (*p* = 0.107) and no main effect for Condition (*p* = 0.353). Similarly to Hb, a main effect for Time was also present (F _[2,22]_=136.499, *p* < 0.001, $\eta_{p}^{2}$ = 0.925). Lastly, percent change in plasma volume also yielded no Condition × Time interaction (*p* = 0.906). Additionally, there was no main effect for Condition (*p* = 0.416); however, a main effect for Time was present (*F*_[1,11]_=110.077, *p* < 0.001, $\eta_{p}^{2}$ = 0.909). Data for all hematologic variables are provided in Table 2. For the metabolic analyte BLa, there was no significant Condition × Time interaction or main effect for Condition; however, there was a main effect for Time (*F*_[2,22]_=121.843, *p* < 0.001, $\eta_{p}^{2}$ = 0.917) as displayed in Figure 3.

**TABLE 2:** Exercise‐induced changes in hematologic variables.

	HL‐RE			LL‐BFR		
	PRE	IP	+5 min	PRE	IP	+5 min
Hemoglobin, g·dL^−1^	14.6 ± 1.2	15.9 ± 0.9	15.4 ± 1.0	14.5 ± 0.7	15.8 ± 0.9	15.4 ± 0.9
Hematocrit, %	47.0 ± 3.4	50.7 ± 3.5	50.0 ± 3.5	45.8 ± 2.0	50.7 ± 2.3	49.4 ± 2.8
% Change in plasma volume	–	−14.5 ± 3.4	−10.2 ± 3.9	–	−15.9 ± 4.8	−11.7 ± 4.6

*Note*: Data presented as x̄ ± SD.

Abbreviations: +5 min, five minutes post‐exercise; HL‐RE, high‐load resistance exercise; IP, immediately post‐exercise; LL‐BFR, low‐load blood flow restricted resistance exercise; PRE, pre‐exercise.

### 3.4 Catecholamines

**FIGURE 4:**
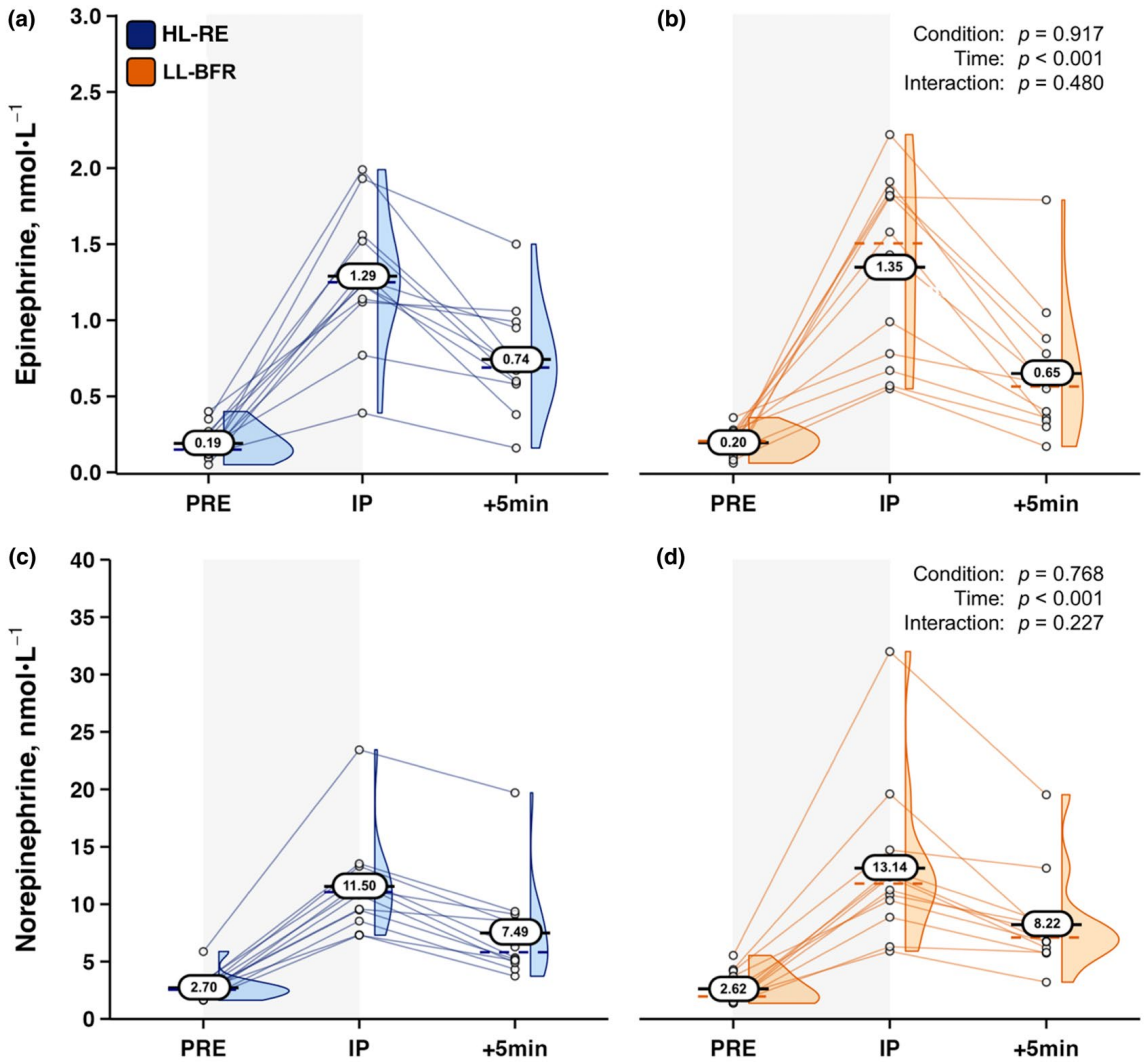
Plasma epinephrine responses to (a) high‐load resistance exercise (HL‐RE) and (b) low‐load blood flow restricted resistance exercise (LL‐BFR), and plasma norepinephrine responses to (c) HL‐RE and (d) LL‐BFR conditions in well‐trained men (*n* = 12). Oval data labels with horizontal lines = means; Dashed colored lines = medians; Histograms are also provided. *p* values from 2 × 3 (Condition × Time) RMANOVAs are overlaid for main effects and interaction effects for each analyte. +5 min, five minutes post‐exercise; IP, immediately post‐exercise; PRE, pre‐exercise.

For the catecholamines, EPI displayed no Condition × Time interaction or main effect for Condition. There was, however, a main effect for Time (*F*_[2,22]_=44.410, *p* < 0.001, $\eta_{p}^{2}$ = 0.801). Log transformed NE also yielded no Condition × Time interaction or Condition main effect, with a significant main effect for Time present (*F*_[2,22]_=321.542, *p* < 0.001, $\eta_{p}^{2}$ = 0.967). Original catecholamine data with corresponding *p* values are shown in Figure 4.

### 3.5 Steroid hormones

**FIGURE 5:**
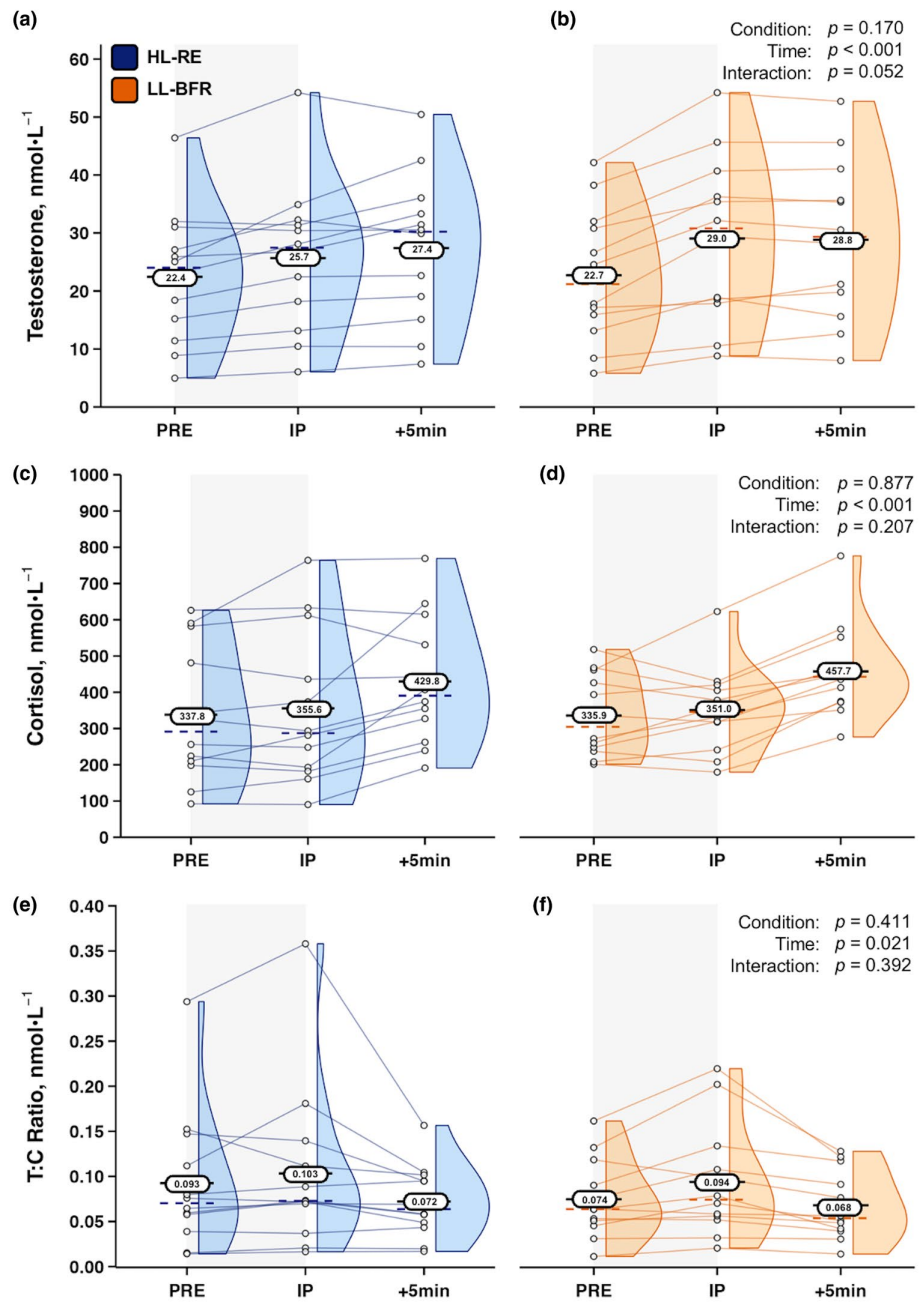
Serum testosterone, cortisol, and testosterone: Cortisol (T/C) ratio responses to high‐load resistance exercise (HL‐RE) (a, c, e), along with responses to low‐load blood flow restricted resistance exercise (LL‐BFR) (b, d, f) in well‐trained men (*n* = 12). Oval data labels with horizontal lines = means; Dashed colored lines = medians; Histograms are also provided. *p* values from 2 × 3 (Condition × Time) RMANOVAs are overlayed for main effects and interaction effects for each variable. +5 min, five minutes post‐exercise; IP, immediately post‐exercise; PRE, pre‐exercise.

For the androgenic steroid T, there was a close, but not significant Condition × Time interaction (*F*_[2,22]_=3.389, *p* = 0.052, $\eta_{p}^{2}$ = 0.236). Further, there was no Condition main effect. However, a main effect for Time was present (*F*_[2,22]_=29.324, *p* < 0.001, $\eta_{p}^{2}$ = 0.727). For the glucocorticoid C, no interaction or Condition main effects were present. Similarly to T, a main effect for Time was found for C (*F*_[2,22]_=15.324, *p* < 0.001, $\eta_{p}^{2}$ = 0.582). When evaluated as a ratio, T/C displayed neither an interaction nor a main effect for Condition; however, a main effect for Time was found (*F*_[2,22]_=4.598, *p* = 0.021, $\eta_{p}^{2}$ = 0.295). Data for both steroid hormones and the ratio metric T/C are displayed in Figure 5.

### 3.6 Growth hormone‐22 kDa

**FIGURE 6:**
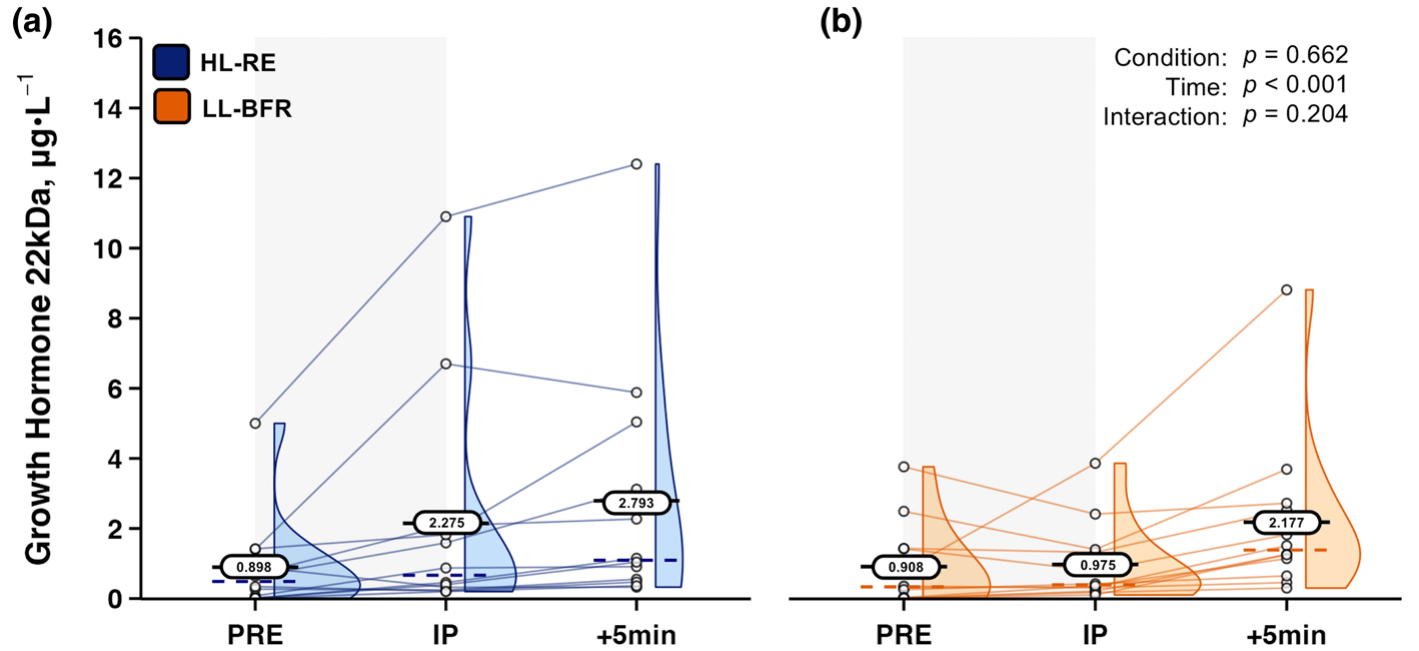
Serum immunofunctional 22‐kilodalton growth hormone responses to (a) high‐load resistance exercise (HL‐RE) and (b) low‐load blood flow restricted resistance exercise (LL‐BFR) in well‐trained men (*n* = 12). Oval data labels with horizontal lines = means; Dashed colored lines = medians; Histograms at each time point also provided. *p* values from 2 × 3 (Condition × Time) repeated‐measures ANOVA are overlayed for main effects and interaction effects. +5 min, five minutes post‐exercise; IP, immediately post‐exercise; PRE, pre‐exercise.

For the immunofunctional GH‐22 kDa, similarly to other analytes, there was no Condition × Time interaction, nor Condition main effect. However, a main effect for Time was found (*F*_[2,22]_ = 13.127, *p* < 0.001, $\eta_{p}^{2}$ = 0.544). GH‐22 kDa data were log transformed for these analyses due to non‐normal data distributions. Original GH‐22 kDa data are shown in Figure 6.

## 4 DISCUSSION

Collectively, the primary findings of the present investigation are that LL‐BFR performed with relatively high pressure (80% AOP) resulted in individuals lifting lower volume‐loads while completing a greater number of total repetitions than the comparative condition HL‐RE, and both protocols, despite differences in relative intensity (e.g., 30% vs. 70% 1RM) and volume‐related metrics, evoked no statistically significant differences in any of the acute exercise‐induced elevations in systemic metabolic and hormonal analyte responses measured. Thus, LL‐BFR appears to be capable of inducing similar endocrine responses to HL‐RE in well resistance‐trained men. Importantly, elevations in venous concentrations of the potent β_2_AR agonist EPI were robust but comparable between conditions, which, in addition to the increases in NE observed, suggests similar sympathetic nervous system activity during both LL‐BFR and HL‐RE. Further, marked blunting in SmO_2_resat was present due to the continuous venous occlusion applied during LL‐BFR, which likely expedited the onset of local muscular fatigue during this protocol. Such findings for this proxy of interset local muscle O_2_ recovery corroborate reports of BFR's ability to hinder SmO_2_resat in well resistance‐trained men in comparison to volume‐matched non‐BFR resistance exercise (Eserhaut et al., 2024).

Previous work has shown various LL‐BFR protocols to be capable of inducing significant acute elevations in the catecholamine NE (Madarame et al., 2010; Takano et al., 2005; Takarada et al., 2000), which aligns with NE responses displayed in Figure 4. While this is representative, in part, of elevated sympathetic nervous system activity, within the context of training related skeletal muscle interactions, EPI is the primary catecholamine of interest due to β_2_AR being the dominant receptor subtype on this type of muscle tissue (Williams et al., 1984). To the best of our knowledge, we believe this to be the first reporting of EPI responses to LL‐BFR relative to HL‐RE in men of a higher resistance training status. Marked, yet not significantly different acute elevations in EPI may be indicative of comparable acute β_2_AR phosphorylation mediated downstream intra‐cellular signaling cascades during the two protocols. The accumulation of the β_2_AR secondary messenger cyclic adenosine monophosphate (cAMP) plays a critical role in intra‐muscular glycogen metabolism (Chasiotis, 1988) and skeletal muscle protein synthesis (Koopman et al., 2010), with similar venous EPI concentrations suggesting similar magnitudes of intra‐muscular glycogen metabolism and protein synthesis may have occurred acutely during and after LL‐BFR and HL‐RE. Indeed, data investigating the effects of graded EPI infusions during steady‐state low‐intensity cycling on the metabolic analyte BLa show a dose–response relationship between greater systemic EPI concentrations and resultant BLa measures, indicating β_2_AR phosphorylation influences skeletal muscle glycogen metabolism during exercise (Turner et al., 1995). While EPI mediated β_2_AR phosphorylation signals for processes that influence reactions involved in skeletal muscle protein synthesis, the outcome of muscle fiber hypertrophy is complex and multifactorial in nature, with many contributory mechanisms (Roberts et al., 2023).

Additional endocrine‐related mechanisms center on the analytes T and GH‐22 kDa, both of which have been investigated extensively within the context of resistance exercise‐induced acute elevations (Nindl et al., 2003; Vingren et al., 2010). LL‐BFR's ability to augment T acutely has received little attention, with data from a cohort of college‐aged untrained men showing LL‐BFR seated leg extensions performed for 6 sets × 15 repetitions at 30% 1RM with comparably high pressures of 70% AOP relative to the present study elevates venous concentrations of T acutely post‐exercise (Yinghao et al., 2021). However, in highly resistance‐trained cohorts, data is scarce, with practical elastic wrap BFR methods applied during low‐load barbell back squats being capable of increasing salivary free T concentrations in American football athletes (Luebbers et al., 2014). Importantly, these LL‐BFR serum T responses have not been compared directly to HL‐RE protocols, especially so in trained men. Provided data suggests acute elevations in the androgenic hormone T are not different between LL‐BFR and a higher‐load non‐BFR failure protocol. It is speculated that acute skeletal muscle androgen receptor activity may also be comparable. The bioactive form of the pituitary gland‐derived peptide GH‐22 kDa has also been shown to increase within venous circulation following LL‐BFR in young untrained males, with higher BFR pressures inducing the most pronounced elevations (Pierce et al., 2006; Takarada et al., 2000; Yinghao et al., 2021). While significant elevations in GH‐22 kDa did occur in well resistance‐trained individuals following LL‐BFR, there was marked variance in responses between individuals evidenced by the provided individual data (see Figure 6). Further, no protocol‐specific differences were observed between LL‐BFR and HL‐RE. Such variance in the GH‐22 kDa response to resistance exercise has been reported before, evidenced by the wide standard error bars shown in multiple other reports suggesting some individuals have very minimal acute elevations and some very pronounced increases post‐resistance exercise (Feldmann et al., 2019; Pierce et al., 2006).

It is possible that extending post‐exercise blood sampling out to +15 and +30 min may have yielded greater GH‐22 kDa concentrations following both protocols for some individuals, as peak concentrations have been reported 15–30 min post‐ LL‐BFR (Madarame et al., 2010; Yinghao et al., 2021) previously. The specific mechanisms of GH‐22 kDa release during LL‐BFR have yet to be fully elucidated. Non‐BFR exercise studies suggest the GH‐22 kDa response may be linked to adrenergic receptor stimulation (Weltman et al., 2000), cholinergic receptor stimulation (Thompson et al., 1993), or possibly systemic metabolite accumulation and subsequent muscle chemoreceptor stimulation by way of type III and IV afferent nerves (Pierce et al., 2006). Additionally, numerous different growth hormone isoforms and dimerization variants exist within venous circulation, each with related, yet distinct physiological roles (Nindl et al., 2003). Whether or not the unique physiological stress imposed by combining venous occlusion and resistance exercise (LL‐BFR) induces differential growth hormone isoform responses has not yet been investigated. Data from aerobically trained men suggest high‐intensity steady‐state cycling is capable of increasing the venous concentrations of numerous growth hormone isoforms, but with isoforms displaying varied rates of clearance from circulation (Wallace et al., 2001). Between‐protocol comparisons of growth hormone isoform responses, specifically within the context of resistance exercise, are currently understudied. Nevertheless, LL‐BFR and HL‐RE bilateral seated leg extension failure protocols both appear capable of acutely elevating the immunofunctional isoform GH‐22 kDa.

Lastly, both LL‐BFR and HL‐RE led to significant increases in serum C concentrations. Given marked, yet comparable EPI responses following both protocols, C data support the notion that similar adrenal stimulation occurred during and after the two different resistance exercise training methods and that LL‐BFR and HL‐RE evoked comparable endocrine stress responses and similar metabolic demands during exercise (Dipla et al., 2021; Fry et al., 1994). The metabolic analyte BLa has displayed significant increases in recreationally active men following bilateral seated leg extension LL‐BFR performed using 30% 1RM with 60% AOP, with BLa concentrations comparable to high‐load (70% 1RM) non‐BFR training (Loenneke et al., 2017). Similar high‐pressure bilateral seated leg extension LL‐BFR protocols have been shown to elevate BLa concentrations to circa 10 mmol·L^−1^ in well resistance‐trained men (Eserhaut et al., 2024). Despite the application of continuous BFR augmenting the BLa response to low‐load resistance exercise, BLa elevations were no different from traditional HL‐RE in the present study with a comparable cohort of resistance‐trained individuals.

### 4.1 Limitations and future recommendations

Our findings should be interpreted within the context of some limitations. First, no a priori power analysis was conducted, and the sample size was constrained by available resources (Lakens, 2022), necessitating caution in the interpretation of our results. Sampling from a larger cohort of subjects using similar within‐subjects designs would aid in determining whether smaller between‐protocol effects exist via increasing statistical power. The within‐subjects design employed and registered dietitian administered 24 h dietary recalls collectively aid in minimizing data variability via eliminating the potential for between‐subject variance and reducing the influence of abnormal dietary patterns on the exercise induced physiological responses. Nevertheless, heightened statistical power may provide more granular insight. Additionally, work measuring EPI responses to LL‐BFR protocols employing lower pressures (e.g., 50%–60% AOP) and/or set termination further from momentary task failure may be a future area of interest as LL‐BFR high pressure failure protocols may impart heightened levels of acute discomfort (Spitz et al., 2021), which may limit routine implementation during moderate‐to‐long term strength and conditioning regimens. Such work would provide valuable insight on the physiological effects of LL‐BFR protocols that may be more tolerable on average.

## 5 CONCLUSION

In closing, LL‐BFR and HL‐RE appear to evoke comparable acute elevations in BLa, T, C, T/C, GH‐22 kDa, EPI, and NE in well resistance trained men (1.8× bodyweight back squat 1RM). The LL‐BFR protocol requires the performance of less volume‐load, with the completion of a greater number of total repetitions across four sets taken to momentary task failure in comparison to HL‐RE. It is possible that one contributory mechanism underpinning continuous BFR's ability to expedite the onset of momentary task failure is the pronounced blunting in SmO_2_resat during interset periods of passive rest, limiting the quantity of available O_2_ within the microcapillaries of exercising skeletal muscle at the start of later sets (e.g., sets 2, 3, and 4). This impaired local recovery during multi‐set LL‐BFR protocols may require individuals to express similar degrees of physical exertion during resistance exercise despite the differences in relative loading (e.g., 30% vs. 70% 1RM) between the two training methods. Thus, when exposure to lighter absolute loads is desired, such as during periods of de‐loading, tapering, or recovery after musculoskeletal injury, practitioners may prescribe LL‐BFR and athletes may achieve similar acute endocrine and BLa responses to that of traditional HL‐RE. Lastly, investigations targeting direct measurement of β_2_AR phosphorylation following LL‐BFR and HL‐RE would expand upon the EPI data reported herewithin, providing further mechanistic insight into LL‐BFR's effects on β_2_AR mediated signaling cascades within skeletal muscle tissue.

## AUTHOR CONTRIBUTIONS

D.A.E., J.M.D., and J.A.P. performed experiments. D.A.E. analyzed data, prepared figures, and drafted manuscript. D.A.E., J.M.D., J.A.P., and A.C.F. conceived and designed research, interpreted results of experiments, edited and revised manuscript, and approved final version of manuscript.

## FUNDING INFORMATION

The article processing charges related to the publication of this article were supported by The University of Kansas (KU) One University Open Access Author Fund sponsored jointly by the KU Provost, KU Vice Chancellor for Research, and KUMC Vice Chancellor for Research and managed jointly by the Libraries at the Medical Center and KU—Lawrence.

## DISCLOSURE

Joseph M. DeLeo owns and operates Science of Rowing, LLC, a digital publication that publishes reviews of rowing research.

## ETHICS STATEMENT

The University of Kansas' institutional review board approved this study (STUDY#00151133) and all subjects provided written informed consent following a thorough briefing on study procedures in accordance with the 1964 Helsinki Declaration.

## Data Availability

The data that support the findings of this study are available from the corresponding author, D. A. E., upon reasonable request.
